# Public health insurance and the risk of cancer-specific mortality in patients with cervical cancer: A Chinese prospective cohort study

**DOI:** 10.3389/fpubh.2023.1121548

**Published:** 2023-03-30

**Authors:** Li Yuan, Haike Lei, Dongling Zou, Baogang Wen, Xiuying Li, Qianjie Xu, Ying Wang, Qi Zhou

**Affiliations:** ^1^Department of Gynecologic Oncology, Chongqing University Cancer Hospital, Chongqing, China; ^2^Chongqing Cancer Multi-omics Big Data Application Engineering Research Center, Chongqing University Cancer Hospital, Chongqing, China; ^3^Department of Health Statistics, School of Public Health, Chongqing Medical University, Chongqing, China

**Keywords:** cervical cancer, cancer specific mortality, public health insurance, prognosis, prospective cohort study

## Abstract

**Objective:**

Cervical cancer has one of the highest incidence and mortality rates of any malignant tumor of the female reproductive tract, and its longer treatment period will place significant financial strain on patients and their families. Little is known about how health insurance policies influence cervical cancer prognosis, particularly in developing countries. The relationship between cervical cancer specific death and cervical cancer all-cause mortality with public health insurance, self-payment rate, and the combined effect of public health insurance and self-payment rate was investigated in this study.

**Materials and methods:**

From 2015 to 2019, a prospective longitudinal cohort study on cervical cancer was carried out in Chongqing, China. We chose 4,465 Chongqing University Cancer Hospital patients who had been diagnosed with cervical cancer between 2015 and 2019. The self-payment rate and public health insurance are taken into account in our subgroup analysis. After applying the inclusion and exclusion criteria, we describe the demographic and clinical traits of patients with various insurance plans and self-payment rates using the chi-square test model. The relationship between cervical cancer patients with various types of insurance, the self-payment rate, and treatment modalities is examined using the multivariate logistic regression model. After applying the inclusion and exclusion criteria, we summarize the demographic and clinical traits of patients with various insurance plans and self-payment rates using the chi-square test model. The association between cervical cancer patients with various types of insurance, the self-payment rate, and treatment modalities is examined using the multivariate logistic regression model. The cumulative hazard ratio of all-cause death and cervical cancer-specific mortality for various insurance types and self-payment rates was then calculated using the Cox proportional hazard model and the competitive risk model.

**Results:**

This study included a total of 3,982 cervical cancer patients. During the follow-up period (median 37.3 months, 95% CI: 36.40–38.20), 774 deaths were recorded, with cervical cancer accounting for 327 of them. Patients who obtained urban employee-based basic medical insurance (UEBMI) had a 37.1% lower risk of all-cause death compared to patients who received urban resident-based basic medical insurance (URBMI) (HRs = 0.629, 95% CI: 0.508–0.779, *p* = 0.001). Patients with a self-payment rate of more than 60% had a 26.9% lower risk of cervical cancer-specific mortality (HRs = 0.731, 95% CI: 0.561–0.952, *p* <0.02).

**Conclusions:**

The National Medical Security Administration should attempt to include the more effective self-paid anti-tumor medications into national medical insurance coverage within the restrictions of restricted medical insurance budget. This has the potential to reduce not only the mortality rate of cervical cancer patients, but also their financial burden. High-risk groups, on the other hand, should promote cervical cancer screening awareness, participate actively in the state-led national cancer screening project and enhance public awareness of HPV vaccine. This has the potential to reduce both cervical cancer patient mortality and the financial burden and impact.

## Introduction

Amazing advancements in cancer treatment have led to better patient outcomes, but these benefits have come at an increasing cost. As the cost of healthcare rises, insurers have moved some of the financial burden to consumers by raising deductibles, copayments, and coinsurance, which has led to an increase in out-of-pocket expenditures (1). Cancer is one of the most expensive diseases to treat in the United States, and with the increased use of chemotherapy, biologic, and other targeted therapies, an increase in the number of cancer survivors, and an increase in the cost of new cancer treatments, these costs are expected to rise in the future (2). All cancer patients indeed bear a crushing burden, those who are particularly susceptible to financial strain bear it more heavily than others. Particularly in developing nations where the healthcare system is underdeveloped and unable to relieve the burden for everyone, cancer patients frequently experience severe financial stress throughout their survivor ship (3). Furthermore, cancer survivors are more likely to report being unable to work due to illness or disability, including more missed work days or additional days spent in bed due to illness. Limitations in ability to work may also reduce employment-based health insurance options and resources to pay for medical care, exacerbating cancer's financial impact (2).

Cervical cancer is the most common malignant tumor among women worldwide. In decade, the incidence rate ranks fourth among female malignant tumors, and even ranks first in some developing countries. Cervical cancer is an important disease that endangers the health and life of Chinese women. Cervical cancer is a growing global burden, especially in developing nations. In 2018, ~570,000 cases of cervical cancer and 311,000 deaths from cervical cancer worldwide (4). The knowledge that main preventable risk factors for cervical cancer include in viral persistence human papilloma virus (HPV) infection and etiology of cervical cancer include smoking habits, high parity, long term oral contraceptive use, and coinfection with other sexually transmitted diseases (5). Cervical cancer incidence and mortality rates have shown a long-term downward trend worldwide since the introduction of cervical cancer screening and the human papilloma virus (HPV) vaccine. Cervical cancer mortality rates in China, on the other hand, have not yet fallen to comparable ideal levels. China accounted for 11.9% of global cervical cancer deaths and 12.3% of cervical cancer DALYs in 2017 due to its large population (6, 7). Cervical cancer is a chronic gynecological disease with long treatment cycle and complex treatment conditions. The high cost of cervical cancer treatment and anticancer drugs not only puts a strain on individuals and families, but also puts a huge financial burden on a country's health system, especially in developing countries where the health system is not prepared to reduce everyone's medical burden (8). There is sufficient evidence that social inequality in health leads to differences in cancer survival rates in developed and developing countries, including China. The existence of public medical insurance system seems to be crucial for a country to achieve universal medical coverage and medical equity. The medical insurance system is a type of insurance that is used to reimburse medical expenses incurred as a result of diseases. Although the global burden of disease study estimates the trend of incidence rate and mortality through modeling, there is still a research gap in its time pattern using data from the real-world cancer registries. Evidence suggests that financial burdens are common, affecting younger and more economically disadvantaged cancer patients disproportionately (9). There are currently few studies on the relationship between the self-payment rate and the prognosis of cancer, with the majority of studies concentrating only on the relationship between financial burdens and the prognosis of cancer (3). Therefore, the purpose of the research was to use a prospective large-scale cohort study of cervical cancer patients diagnosed in Chongqing to explore the relationship among cervical cancer specific mortality and its insurance type, self-payment rate, and the combined impact of insurance type and self-payment rate in Chongqing.

## Materials and methods

### Study population

The follow-up database at the Chongqing University Cancer Hospital, which contains almost all cervical cancer patients in Chongqing since 2015, is the foundation of this prospective cohort study. We identified 4,465 cervical cancer patients between May 1, 2015 and December 12, 2019, and we gathered pertinent demographic (gender, race, marital status, occupation, age at diagnosis, date of diagnosis), clinical characteristics (cancer stage, pathological type, HPV infection status, human albumin, lymphocytes, neutrophils), treatment methods (surgery, targeted therapy, immunotherapy, radiotherapy, chemotherapy), and follow-up information for each of them.

### Inclusion and exclusion criteria

The study's inclusion criteria were as follows (1) patients with new onset cervical cancer diagnosed by the Chongqing University Cancer Hospital, and the main treatment occurred in the hospital; (2) the patient's basic information, medical expense information, and clinical data are all complete, including the patient's clinical diagnosis, pathological data, treatment plan, and follow-up information.

The study's exclusion criteria were as follows (1) non-new cervical cancer patients, or patients who did not receive standard treatment; (2) lack of important information such as medical insurance information and medical expense information; (3) there are no follow-up records.

### Follow-up information

All patients' follow-up information is actively tracked through medical records and telephone contact until death or May 31, 2022, whichever comes first. As far as possible, the potential cause of death should be determined from the medical record or informed by immediate family members.

### Health insurance

The urban basic medical insurance system in China has primarily consisted of two public medical insurance systems: urban employee-based basic medical insurance (UEBMI) and urban resident-based basic medical insurance (URBMI), aims to make it easier for individuals to obtain medical treatment (10, 11). The construction of urban employee-based basic medical insurance (UEBMI) began in the late 1990s, and its insured objects are urban workers. All urban employers and their employees and retirees must participate in the insurance. From October, 2007, students, children and other non-working urban residents covered by the urban resident-based basic medical insurance (URBMI) (12). The medical insurance system is an important guarantee to reduce the family economic burden of patients with chronic diseases and ensure their timely access to the required health services. In 2011, the coverage rate of China's basic medical insurance exceeded 95%, achieving universal medical insurance in terms of coverage rate. We classified insurance types into UEBMI and URBMI schemes, respectively. The total cost of this study refers to the total cost of all costs incurred during the observation period, beginning with the new diagnosis of cervical cancer and ending with the completion of all treatment. The self-paying rate is defined as the cost that patients and their families pay themselves to receive health care services divided by the total cost. For the same disease, everyone has a different self-payment rate, which is influenced by factors like age, employment status, and disease management strategy, among others. In this study, information about the type of insurance and self-payment rate is recorded in the hospital information system (HIS). We classified patients based on whether their reimbursement rate was less than (0%−60%) or greater than (60%−100%) the median.

### Cervical cancer-specific and overall mortality

This study primarily uses outpatient, inpatient, and HIS to query and match patients' survival statuses, as well as telephone, We-Chat, SMS, and other methods for active follow-up until death or May 31, 2022, whichever comes first. We obtain the cause of death of patients by reporting their deaths or actively following up their families. The primary outcome in this study was cervical cancer specific mortality, and the secondary outcome was overall mortality.

### Statistical analysis

First, this study makes a descriptive analysis of baseline characteristics. The demographic and clinical characteristics of patients with various insurance types and reimbursement rates are described. The counting data is presented in frequency and percentage form. We use multiple logistic regression to examine the relationship between different types of insurance, self-payment rate, and cervical cancer treatment (surgery, radiotherapy, chemotherapy, targeted therapy, immunotherapy, and so on). Next, this study compared the risk of cervical cancer specific death and overall mortality in two types of insurance and two groups of self-payment rates. We use the patients receiving URBMI and the patients with low self-payment rates as the reference groups for the insurance type. We use the Cox regression model to determine the risk ratio and 95% confidence interval for overall and cervical cancer-specific mortality in the self-payment rate group. In addition, we used a competitive risk model to calculate and plot the cumulative risk of cervical cancer specific death and overall mortality. Finally, we examined the correlation between every 10% increase in self-paying rate and the risk of both cervical cancer-specific mortality and all-cause mortality in order to better understand the interaction between insurance type and self-paying rate.

This study made adjustments for demographic factors in model A, such as diagnostic age, race, marital status, and occupation. In model B, we also made adjustments based on the clinical characteristics, such as the cervical cancer diagnosis date, cancer stage, and pathological diagnosis, HPV infection status, human albumin, lymphocytes, neutrophils, etc. Additional changes were made to the treatment modalities in model C, including, where appropriate, surgery, targeted therapy, immunotherapy, radiotherapy, and chemotherapy.

The model includes all variables. All data were analyzed using SAS 9.4 statistical software (version 9.4; SAS Institute Inc., Cary, North Carolina) and R software (version 4.0.2; R foundation for statistical computing, Vienna, Austria). *p* < 0.05 was statistically significant.

## Results

### Characteristics of subjects

We excluded 438 patients with missing clinical information and 45 patients with no insurance information or out-of-pocket expenses based on the inclusion and exclusion criteria. In the last cohort, there were 3,982 patients with cervical cancer. They are mostly Han, with only 98 ethnic minorities. Among the included subjects, 2,968 received URBMI and the remaining 1,114 received UEBMI. The median self-paying rates of URBMI and UEBMI were 62.54% and 39.72% respectively. After grouping the self-payment rate, the self-payment rate of 2,124 patients were <60%, and the other 1,858 patients had a higher self-payment rate. Patients receiving URBMI were younger (60.11 ± 9.82 vs. 63.19 ± 10.54, *p* < 0.0001). Among the different types of insurance, there was difference in marital status, the age of diagnosis date, occupation, ethnic group, pathological, stage, HPV16 infection status, serum albumin, neutrophil granulocyte, lymphocyte and other variables were statistically significant (*p* < 0.05). There was a statistically significant difference in the age of diagnosis date, occupation, stage, surgery, radiotherapy, chemotherapy, targeted, immunity, and lymphocyte in the self-payment rate group (*p* < 0.05). Table 1 displays the detailed results.

**Table 1:** Characteristics of patients with cervical cancer by insurance type and self-paying rate.

		**By insurance type**			**By self-paying rate**		
	**All (*****N*** = **3,982)**	**URBMI (*****N*** = **2,868)**	**UEBMI (*****N*** = **1,114)**	***p***	≤ **60%**	>**60%**	***p***
	***N*** **(%)**	***N*** **(%)**	***N*** **(%)**		**(*****N*** = **2,124)** ***N*** **(%)**	**(*****N*** = **1,858)** ***N*** **(%)**	
**Age at diagnosis**				0.002			<0.001
≤ 50	1,750 (43.95)	1,265 (44.11)	485 (43.54)		907 (42.70)	843 (45.37)	
51–60	1,286 (32.30)	960 (33.47)	326 (29.26)		656 (30.89)	630 (33.91)	
>60	946 (23.76)	643 (22.42)	303 (27.20)		561 (26.41)	385 (20.72)	
**Marital status**				<0.001			0.770
Married	3,613 (90.73)	2,636 (91.91)	977 (87.70)		1,924 (90.58)	1,689 (90.90)	
Unmarried/divorced/widowed/other	369 (9.27)	232 (8.09)	137 (12.30)		200 (9.42)	169 (9.10)	
**Occupation**				<0.001			<0.001
Company employees/workers	686 (17.23)	394 (13.74)	292 (26.21)		434 (20.43)	252 (13.56)	
Self-employed/unemployed/freelancers/farmer	1,565 (39.30)	1,314 (45.82)	251 (22.53)		643 (30.27)	922 (49.62)	
Retired (retired) retired personnel/civil servants/professional and technical personnel	441 (11.07)	168 (5.86)	273 (24.51)		320 (15.07)	121 (6.51)	
Other professional	1,290 (32.40)	992 (34.59)	298 (26.75)		727 (34.23)	563 (30.30)	
**Ethnic group**				<0.001			0.439
Han	3,884 (97.54)	2,778 (96.86)	1,106 (99.28)		2,076 (97.74)	1,808 (97.31)	
Minority	98 (2.46)	90 (3.14)	8 (0.72)		48 (2.26)	50 (2.69)	
**Histopathological grading**				<0.001			0.195
1	3,468 (87.09)	2,539 (88.53)	929 (83.39)		1,853 (87.24)	1,615 (86.92)	
2	390 (9.79)	250 (8.72)	140 (12.57)		197 (9.27)	193 (10.39)	
3	124 (3.11)	79 (2.75)	45 (4.04)		74 (3.48)	50 (2.69)	
**Stage**				0.001			<0.001
I	1,358 (34.10)	931 (32.46)	427 (38.33)		642 (30.23)	716 (38.54)	
II	1,246 (31.29)	901 (31.42)	345 (30.97)		770 (36.25)	476 (25.62)	
III	1,108 (27.83)	839 (29.25)	269 (24.15)		582 (27.40)	526 (28.31)	
IV	270 (6.78)	197 (6.87)	73 (6.55)		130 (6.12)	140 (7.53)	
***N***				0.226			0.548
≤ 6.46	3,541 (89.26)	2,540 (88.87)	1,001 (90.26)		1,886 (88.96)	1,655 (89.60)	
>6.46	426 (10.74)	318 (11.13)	108 (9.74)		234 (11.04)	192 (10.40)	
**LY**				0.010			0.032
≤ 0.89	527 (13.28)	405 (14.17)	122 (11.00)		305 (14.39)	222 (12.02)	
>0.89	3,440 (86.72)	2,453 (85.83)	987 (89.00)		1,815 (85.61)	1,625 (87.98)	
**Alb**				0.189			0.255
≤ 33.67	125 (3.16)	97 (3.41)	28 (2.53)		60 (2.84)	65 (3.53)	
>66.67	3,830 (96.84)	2,751 (96.59)	1,079 (97.47)		2,052 (97.16)	1,778 (96.47)	
**Hpv16**				<0.001			0.213
Negative	2,373 (59.59)	1,653 (57.64)	720 (64.63)		1,246 (58.66)	1,127 (60.66)	
Positive	1,609 (40.41)	1,215 (42.36)	394 (35.37)		878 (41.34)	731 (39.34)	
**Hpv18**				0.419			0.140
Negative	3,649 (91.64)	2,635 (91.88)	1,014 (91.02)		1,933 (91.01)	1,716 (92.36)	
Positive	333 (8.36)	233 (8.12)	100 (8.98)		191 (8.99)	142 (7.64)	
**Hpv52**				0.541			0.498
Negative	3,514 (88.25)	2,537 (88.46)	977 (87.70)		1,867 (87.90)	1,647 (88.64)	
Positive	468 (11.75)	331 (11.54)	137 (12.30)		257 (12.10)	211 (11.36)	
**Hpv58**				0.807			0.779
Negative	3,605 (90.53)	2,599 (90.62)	1,006 (90.31)		1,926 (90.68)	1,679 (90.37)	
Positive	377 (9.47)	269 (9.38)	108 (9.69)		198 (9.32)	179 (9.63)	
**Surgery**				0.666			<0.001
No	1,961 (49.25)	1,419 (49.48)	542 (48.65)		1,155 (54.38)	806 (43.38)	
Yes	2,021 (50.75)	1,449 (50.52)	572 (51.35)		969 (45.62)	1,052 (56.62)	
**Radiotherapy**				0.378			<0.001
No	2,593 (65.12)	1,880 (65.55)	713 (64.00)		1,584 (74.58)	1,009 (54.31)	
Yes	1,389 (34.88)	988 (34.45)	401 (36.00)		540 (25.42)	849 (45.69)	
**Chemotherapy**				0.273			<0.001
No	2,589 (65.02)	1,880 (65.55)	709 (63.64)		1,582 (74.48)	1,007 (54.20)	
Yes	1,393 (34.98)	988 (34.45)	405 (36.36)		542 (25.52)	851 (45.80)	
**Targeted**				0.007			<0.001
No	3,786 (95.08)	2,744 (95.68)	1,042 (93.54)		2,051 (96.56)	1,735 (93.38)	
Yes	196 (4.92)	124 (4.32)	72 (6.46)		73 (3.44)	123 (6.62)	
**Immunity**				0.377			<0.001
No	3,949 (99.17)	2,847 (99.27)	1,102 (98.92)		2,122 (99.91)	1,827 (98.33)	
Yes	33 (0.83)	21 (0.73)	12 (1.08)		2 (0.09)	31 (1.67)	

Table 2 shows the associations of demographic factors, the clinical characteristics, and treatment types with cervical cancer-specific mortality and all cause death. Among the included subjects, 774 patients with all-cause mortality, including 327 cervical cancer-specific deaths. Among the all-cause mortality group, there was difference in the age of diagnosis date, occupation, histopathological grading, stage, neutrophil granulocyte, lymphocyte, serum albumin, HPV52 infection status, HPV58 infection status, surgery, radiotherapy, chemotherapy and targeted were statistically significant (*p* < 0.05). There was a statistically significant difference in the occupation, histopathological grading, stage, neutrophil granulocyte, lymphocyte, serum albumin, surgery, radiotherapy, chemotherapy and targeted in the cervical cancer-specific mortality group (*p* < 0.05).

**Table 2:** Associations of demographic factors, the clinical characteristics, and treatment types with cervical cancer-specific mortality and all cause death.

		**Overall mortality**			**Cervical cancer-specific mortality**		
	**All (*****N*** = **3,982)**	**Others (*****N*** = **3,208)**	**Death (*****N*** = **774)**	***p***	**Others (*****N*** = **3,655)**	**Death (*****N*** = **327)**	***p***
	***N*** **(%)**	***N*** **(%)**	***N*** **(%)**		***N*** **(%)**	***N*** **(%)**	
**Age at diagnosis**				<0.001			0.059
≤50	1,750 (43.95)	1,468 (45.76)	282 (36.43)		1,619 (44.30)	131 (40.06)	
51–60	1,286 (32.30)	1,056 (32.92)	230 (29.72)		1,185 (32.42)	101 (30.89)	
>60	946 (23.76)	684 (21.32)	262 (33.85)		851 (23.28)	95 (29.05)	
**Marital status**				0.975			0.051
Married	3,613 (90.73)	2,910 (90.71)	703 (90.83)		3,306 (90.45)	307 (93.88)	
Unmarried/divorced/widowed/other	369 (9.27)	298 (9.29)	71 (9.17)		349 (9.55)	20 (6.12)	
**Occupation**				<0.001			<0.001
Company employees/workers	686 (17.23)	582 (18.14)	104 (13.44)		638 (17.46)	48 (14.68)	
Self-employed/unemployed/freelancers/farmer	1,565 (39.30)	1,310 (40.84)	255 (32.95)		1,472 (40.27)	93 (28.44)	
Retired (retired) retired personnel/civil servants/professional and technical personnel	441 (11.07)	338 (10.54)	103 (13.31)		393 (10.75)	48 (14.68)	
Other professional	1,290 (32.40)	978 (30.49)	312 (40.31)		1,152 (31.52)	138 (42.20)	
**Ethnic group**				0.250			0.343
Han	3,884 (97.54)	3,134 (97.69)	750 (96.90)		3,562 (97.46)	322 (98.47)	
Minority	98 (2.46)	74 (2.31)	24 (3.10)		93 (2.54)	5 (1.53)	
**Histopathological grading**				0.042			0.001
1	3,468 (87.09)	2,805 (87.44)	663 (85.66)		3,201 (87.58)	267 (81.65)	
2	390 (9.79)	314 (9.79)	76 (9.82)		350 (9.58)	40 (12.23)	
3	124 (3.11)	89 (2.77)	35 (4.52)		104 (2.85)	20 (6.12)	
**Stage**				<0.001			<0.001
I	1,358 (34.10)	1,241 (38.68)	117 (15.12)		1,299 (35.54)	59 (18.04)	
II	1,246 (31.29)	1,017 (31.70)	229 (29.59)		1,157 (31.66)	89 (27.22)	
III	1,108 (27.83)	832 (25.94)	276 (35.66)		999 (27.33)	109 (33.33)	
IV	270 (6.78)	118 (3.68)	152 (19.64)		200 (5.47)	70 (21.41)	
***N***				<0.001			0.001
≤ 6.46	3,541 (89.26)	2,903 (90.86)	638 (82.64)		3,269 (89.78)	272 (83.44)	
>6.46	426 (10.74)	292 (9.14)	134 (17.36)		372 (10.22)	54 (16.56)	
**LY**				<0.001			<0.001
≤ 0.89	527 (13.28)	341 (10.67)	186 (24.09)		458 (12.58)	69 (21.17)	
>0.89	3,440 (86.72)	2,854 (89.33)	586 (75.91)		3,183 (87.42)	257 (78.83)	
**Alb**				<0.001			0.000
≤ 33.67	125 (3.16)	77 (2.42)	48 (6.24)		103 (2.84)	22 (6.77)	
>66.67	3,830 (96.84)	3,109 (97.58)	721 (93.76)		3,527 (97.16)	303 (93.23)	
**Hpv16**				0.245			0.310
Negative	2,373 (59.59)	1,897 (59.13)	476 (61.50)		2,169 (59.34)	204 (62.39)	
Positive	1,609 (40.41)	1,311 (40.87)	298 (38.50)		1,486 (40.66)	123 (37.61)	
**Hpv18**				0.261			1.000
Negative	3,649 (91.64)	2,948 (91.90)	701 (90.57)		3,349 (91.63)	300 (91.74)	
Positive	333 (8.36)	260 (8.10)	73 (9.43)		306 (8.37)	27 (8.26)	
**Hpv52**				0.002			0.050
Negative	3,514 (88.25)	2,805 (87.44)	709 (91.60)		3,214 (87.93)	300 (91.74)	
Positive	468 (11.75)	403 (12.56)	65 (8.40)		441 (12.07)	27 (8.26)	
**Hpv58**				0.002			0.095
Negative	3,605 (90.53)	2,881 (89.81)	724 (93.54)		3,300 (90.29)	305 (93.27)	
Positive	377 (9.47)	327 (10.19)	50 (6.46)		355 (9.71)	22 (6.73)	
**Surgery**				<0.001			<0.001
No	1,961 (49.25)	1,453 (45.29)	508 (65.63)		1,752 (47.93)	209 (63.91)	
Yes	2,021 (50.75)	1,755 (54.71)	266 (34.37)		1,903 (52.07)	118 (36.09)	
**Radiotherapy**				<0.001			<0.001
No	2,593 (65.12)	1,938 (60.41)	655 (84.63)		2,299 (62.90)	294 (89.91)	
Yes	1,389 (34.88)	1,270 (39.59)	119 (15.37)		1,356 (37.10)	33 (10.09)	
**Chemotherapy**				<0.001			<0.001
No	2,589 (65.02)	1,931 (60.19)	658 (85.01)		2,294 (62.76)	295 (90.21)	
Yes	1,393 (34.98)	1,277 (39.81)	116 (14.99)		1,361 (37.24)	32 (9.79)	
**Targeted**				<0.001			<0.001
No	3,786 (95.08)	3,070 (95.70)	716 (92.51)		3,498 (95.70)	288 (88.07)	
Yes	196 (4.92)	138 (4.30)	58 (7.49)		157 (4.30)	39 (11.93)	
**Immunity**				0.009			0.159
No	3,949 (99.17)	3,175 (98.97)	774 (100.00)		3,622 (99.10)	327 (100.00)	
Yes	33 (0.83)	33 (1.03)	0 (0.00)		33 (0.90)	0 (0.00)	

### Health insurance and cervical cancer treatments

**Figure 1:**
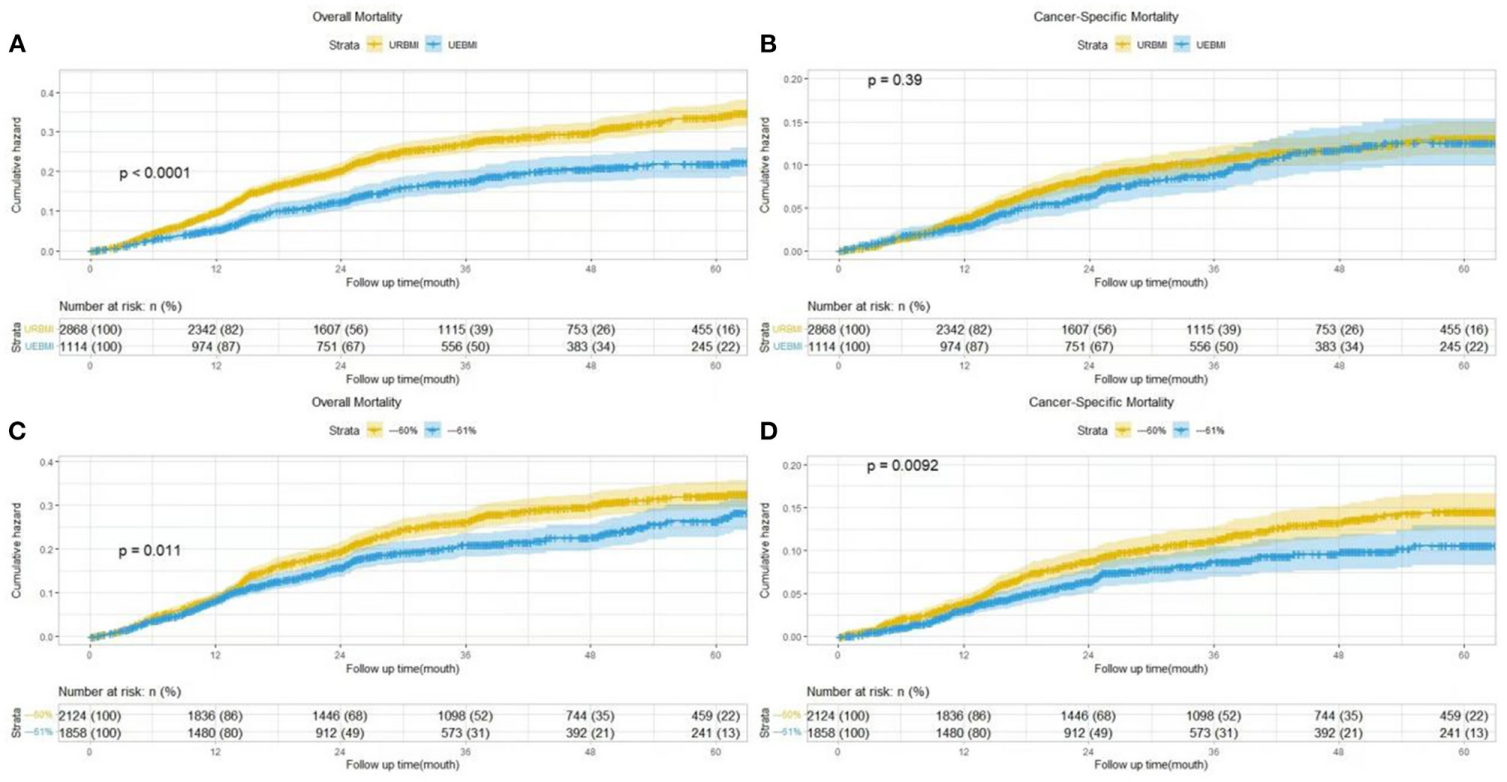
Cumulative hazard of cervical cancer specific or overall mortality by insurance type **(A, B)** and self-paying rate **(C, D)**.

Table 3 shows the relationship between different types of medical insurance, self-payment rate groups and cervical cancer treatment models (including surgery, radiotherapy, chemotherapy, targeted therapy and immunotherapy). As a result of the treatment mode, insurance types and self-payment rate groups are independent variables in the model, which also includes other adjustment variables. After adjusting for pathological diagnosis, cervical cancer stage, date of diagnosis, age at diagnosis, gender, ethnicity, marital status and occupation, patients receiving UEBMI are statistically significantly more likely to select surgery, targeted therapy, immunity therapy, radiotherapy and chemotherapy. Compared to patients with <60% self-paying, patients with higher self-paying tend to prefer surgery, radiotherapy, targeted therapy, immunity therapy, and chemotherapy. The median follow-up time was 37.3 months (95% CI: 36.40–38.20) during the follow-up period. 3,982 deaths were observed, 774 died of cervical cancer. According to Figure 1, compared to patients who received UEBMI and had a self-paying rate, patients who received URBMI and had a self-paying rate had a higher cumulative hazard rate for cervical cancer. When demographic characteristics were controlled for, the risk of all-cause mortality was reduced by 37.1% (95% CI: 0.508–0.779, *p* < 0.001) for patients receiving UEBMI compared to patients receiving URBMI. The complete results are shown in Table 4. The risk of cervical cancer-specific mortality increased for patients receiving UEBMI by 8.3% after adjusting for clinical features and cervical cancer alternative therapies (HRs = 1.083, 95% CI: 0.811–1.444, *p* = 0.590), whereas the risk of all-cause death decreased by 29.4% (HRs = 0.706, 95% CI: 0.565–0.882, *p* = 0.002). Patients with a higher self-paying rate had a 27% lower risk of all-cause death (HRs = 0.73, 95% CI: 0.604–0.883, *p* = 0.001) and a 26.9% lower risk of cervical cancer-specific mortality (HRs = 0.731, 95% CI: 0.561–0.952, *p* = 0.02) compared to patients with an insufficient self-paying rate.

**Table 3:** Associations of insurance type and self-paying rate with treatment type.

	**Model A**		**Model B**		**Model C**	
	**OR (95%CI)**	***p***	**OR (95%CI)**	***p***	**OR (95%CI)**	***p***
**Surgery**						
**Insurance type**						
URBMI	1		1		1	
UEBMI	1.073 (0.923–1.247)	0.360	0.974 (0.83–1.143)	0.748	0.992 (0.844–1.164)	0.918
**Self-paying rate**						
≤ 60%	1		1		1	
>60%	1.523 (1.337–1.736)	<0.001	1.413 (1.23–1.624)	<0.001	1.436 (1.248–1.651)	<0.001
**Radiotherapy**						
**Insurance type**						
URBMI	1		1		1	
UEBMI	1.533 (1.298–1.809)	<0.001	1.566 (1.322–1.855)	<0.001	1.583 (1.335–1.877)	<0.001
**Self-paying rate**						
≤ 60%	1		1		1	
>60%	2.118 (1.837–2.442)	<0.001	2.236 (1.931–2.59)	<0.001	2.269 (1.958–2.63)	<0.001
**Chemotherapy**						
**Insurance type**						
URBMI	1		1		1	
UEBMI	1.531 (1.298–1.806)	<0.001	1.556 (1.314–1.841)	<0.001	1.576 (1.33–1.867)	<0.001
**Self-paying rate**						
≤ 60%	1		1		1	
>60%	2.127 (1.845–2.451)	<0.001	2.236 (1.931–2.588)	<0.001	2.272 (1.961–2.632)	<0.001
**Targeted**						
**Insurance type**						
URBMI	1		1		1	
UEBMI	1.538 (1.11–2.129)	0.001	1.618 (1.157–2.263)	0.005	1.618 (1.156–2.265)	0.005
**Self-paying rate**						
≤ 60%	1		1		1	
>60%	1.975 (1.452–2.687)	0.001	2.056 (1.503–2.812)	<0.001	2.06 (1.505–2.819)	<0.001
**Immunity**						
**Insurance type**						
URBMI	1		1		1	
UEBMI	2.316 (1.1–4.876)	0.027	2.797 (1.296–6.034)	0.009	2.951 (1.352–6.443)	0.007
**Self-paying rate**						
≤ 60%	1		1		1	
>60%	14.457 (3.415–61.195)	<0.001	14.877 (3.509–63.069)	<0.001	15.231 (3.583–64.748)	<0.001

Model A, we adjusted for demographic factors, including age at diagnosis, sex, ethnicity, marital status, and occupation.

Model B, based on model A, was also adjusted according to the clinical characteristics, including the date of diagnosis of cervical cancer, cancer stage, and pathological diagnosis.

Model C, based on model B, we additionally adjusted for treatment types, whenever applicable, including surgery, targeted therapy, immunity therapy, radiotherapy, and chemotherapy.

**Table 4:** Associations of insurance type and self-paying rate with cervical cancer-specific mortality and all-cause death.

	**Number of patients**	**Number of events**	**Rate**	**Model A**		**Model B**		**Model C**	
				**HR (95%CI)**	***p***	**HR (95%CI)**	***p***	**HR (95%CI)**	***p***
**Overall mortality**									
**Insurance type**									
URBMI	2,868	604	7.92	1.000		1.000		1.000	
UEBMI	1,114	170	4.81	0.629 (0.508–0.779)	< 0.001	0.632 (0.51–0.784)	<0.001	0.706 (0.565–0.882)	0.002
**Self-paying rate**									
≤ 60%	2,124	486	7.26	1.000		1.000		1.000	
>60%	1,858	288	6.45	0.615 (0.514–0.737)	<0.001	0.609 (0.508–0.73)	<0.001	0.73 (0.604–0.883)	0.001
**Cervical cancer-specific mortality**									
**Insurance type**									
URBMI	2,868	234	3.07	1.000		1.000		1.000	
UEBMI	1,114	93	2.63	0.974 (0.737–1.286)	0.852	0.98 (0.742–1.295)	0.887	1.083 (0.811–1.444)	0.590
**Self-paying rate**									
≤ 60%	2,124	214	3.20	1.000		1.000		1.000	
>60%	1,858	113	2.53	0.595 (0.462–0.766)	<0.001	0.59 (0.458–0.76)	<0.001	0.731 (0.561–0.952)	0.020

Model A, we adjusted for demographic factors, including age at diagnosis, sex, ethnicity, marital status, and occupation.

Model B, based on model A, was also adjusted according to the clinical characteristics, including the date of diagnosis of cervical cancer, cancer stage, and pathological diagnosis.

Model C, based on model B, we additionally adjusted for treatment types, whenever applicable, including surgery, targeted therapy, immunity therapy, radiotherapy, and chemotherapy.

### Joint effect of insurance type and reimbursement rate

Table 5 shows the joint effect of insurance type and self-paying rate. The risk of all-cause mortality for patients receiving URBMI was reduced by 40.5% for every 10% increase in the self-paying rate (HRs = 0.595, 95% CI: 0.548–0.647, *p* < 0.001), and the risk of all-cause mortality for patients receiving UEBMI was reduced by 24.7% for every 10% increase in the self-paying rate (HRs = 0.753, 95% CI: 0.674–0.842, *p* < 0.001). Similar outcomes were observed in cervical-specific death. The risk of cervical-specific death was decreased by 34% for patients receiving URBMI (HRs = 0.66, 95% CI: 0.593–0.734, *p* < 0.001) and by 24.9% for patients receiving UEBMI (HRs = 0.751, 95% CI: 0.654–0.861, *p* < 0.001) for every 10% increase in the self-paying rate.

**Table 5:** Association of every 10% insurance self-paying rate increase with cervical cancer-specific mortality and all-cause death.

	**Number of patients**	**Number of events**	**Rate**	**Model A**		**Model B**		**Model C**	
				**HR (95% CI)**	***p***	**HR (95% CI)**	***p***	**HR (95% CI)**	***p***
**Overall mortality**									
Insurance type	3,982	774	6.93	0.232 (0.176–0.307)	<0.001	0.232 (0.175–0.308)	<0.001	0.278 (0.207–0.373)	<0.001
URBMI	2,868	604	7.92	0.595 (0.548–0.647)	<0.001	0.593 (0.546–0.645)	<0.001	0.621 (0.571–0.676)	<0.001
UEBMI	1,114	170	4.81	0.753 (0.674–0.842)	<0.001	0.780 (0.703–0.865)	<0.001	0.874 (0.767–0.996)	0.044
**Cervical cancer-specific mortality**									
Insurance type	3,982	327	2.93	0.423 (0.297–0.602)	<0.001	0.424 (0.297–0.604)	<0.001	0.519 (0.357–0.756)	<0.001
URBMI	2,868	234	3.07	0.66 (0.593–0.734)	<0.001	0.66 (0.594–0.735)	<0.001	0.706 (0.635–0.786)	<0.001
UEBMI	1,114	93	2.63	0.751 (0.654–0.861)	<0.001	0.749 (0.653–0.86)	<0.001	0.820 (0.688–0.979)	0.028

Model A, we adjusted for demographic factors, including age at diagnosis, sex, ethnicity, marital status, and occupation.

Model B, based on model A, was also adjusted according to the clinical characteristics, including the date of diagnosis of cervical cancer, cancer stage, and pathological diagnosis.

Model C, based on model B, we additionally adjusted for treatment types, whenever applicable, including surgery, targeted therapy, immunity therapy, radiotherapy, and chemotherapy.

## Discussion

Our finding analyzes the relationship between specific deaths and all-cause death in Chongqing cervical cancer patients and insurance types and self-payment rate used the HIS and follow-up database. Based on 3,982 patients with cervical cancer treated in Chongqing cancer hospital from 2015 to 2019, subgroup analysis was conducted according to the type of insurance and self-payment ratio. Our results showed cervical cancer-specific deaths associated with occupation, histopathological grading, stage, neutrophil granulocyte, lymphocyte, serum albumin, surgery, radiotherapy, chemotherapy and targeted. Our results suggest that a higher self-payment ratio was associated with a significantly lower risk of cervical cancer specific death. We discovered that in the study of the joint effect of medical insurance type and self-paying rate, every 10% increase in URBMI self-payment rate reduces the risk of cervical cancer-specific mortality by 34%, while every 10% increase in UEBMI self-payment rate reduces the risk of cervical cancer-specific mortality by 24.9%.

With the ongoing research of anti-cancer treatment, particularly the development of new targeted therapeutic drugs, many cervical cancer patients who were previously “sentenced to death” have gained hope for life, improved their quality of life, and some has been contained. However, new anticancer drugs are typically expensive, and the majority of them are not covered by China's public medical insurance, limiting drug accessibility (13). Based on GOG-240, the US Food and Drug Administration (FDA) approved the anti-angiogenic drug bevacizumab in combination with chemotherapy in 2014 for advanced and metastatic cervical cancer. The overall survival rate, however, was only extended by 3.5 months (14). Because there is no effective treatment for advanced and recurrent cervical cancer, the 5-year survival rate is <20%, and the use of immunotherapy in cervical cancer was first investigated in this group of patients as well. The KEYNOTE-826 clinical trial discovered that treating PD-L1-positive recurrent metastatic cervical cancer with pembrolizumab in combination with chemotherapy improved overall patient survival (15). One of the changes in the 2020 NCCN cervical cancer guidelines is the addition of pembrolizumab to second-line combination therapy in PD-L1-positive recurrent metastatic cervical cancer (16). If cervical cancer patients, particularly those with recurrent or metastatic cancer, want to improve their survival status, it must come at a high cost. This explains the increase in self-paying patients with cervical cancer in this study as well as the decreased mortality risk. Future cancer disease preventive and treatment strategies should be developed at the person level based on genomic characteristics (17). Precision medicine aims to maximize the quality of healthcare by individualizing the healthcare process to each patient's unique health status (18).

According to this study, increasing the proportion of self-paid expenses for cervical cancer treatment may decrease the risk of death. Cervical cancer patients and their families hope to reduce mortality, but they must also deal with significant economic and psychological stress. Despite the fact that China has achieved universal medical insurance, the basic medical insurance system for various groups remains in a state of separate financing and overall management. The financing and security levels of various types of basic medical insurance vary greatly across the country. Inclusion of a large number of high-value anti-cancer drugs without first carefully designing a scientific and orderly financing and service system will result in medical insurance waste or penetration. It is especially important for the state to plan the financing channels and methods uniformly, as well as to ensure that the supply is informed, in order to make the best use of medical insurance funds. Setting up an independent pharmacoeconomic evaluation department and a perfect evaluation system, as well as prioritizing high-value anti-cancer drugs with incremental costs and good effects within the scope of public security, are all challenges that China must face in order to promote the accessibility of high-value anti-cancer drugs. Fortunately, this phenomenon has been discovered by the Chinese government. Since 2009, the Chinese government has issued many policies to reduce the economic burden of cervical cancer patients (13). After anti-tumor drugs were gradually included in the medical insurance catalog, patients' financial burden decreased significantly, patients' affordability improved significantly.

The rational use of anti-neoplastic drugs can not only improve therapeutic benefits, but also reduce the economic pressure on patients and the national medical insurance system. It is well known that more than 90% of cervical cancer is caused by persistent infection of high-risk human papilloma virus (HPV). Inoculating HPV vaccine (also known as “cervical cancer vaccine”) is the most effective way to prevent cervical cancer (19). Previously, China's HPV vaccine could not be reimbursed before. In 2022, provinces and cities at all levels of the country will gradually be included in the proportion of medical insurance reimbursement. This policy measure can effectively reduce the incidence of cervical cancer. This study has several advantages because the data was obtained from the cancer hospital follow-up database and the hospital HIS system. This is the first time in the last ten years that the relationship between cervical cancer patients and costs has been studied in Southwest China.

Furthermore, this research suggest that underinsured patients face a higher risk of cervical cancer-specific mortality in China. Our finding can provide specific insights to the Chongqing Medical Insurance Bureau. However, there are some limitations to our study that should be considered. To begin, the cost of treating cervical cancer patients in this study is only incurred in the hospital and cannot be estimated outside of the hospital. Second, this study only included patients from Chongqing University cancer hospitals, not patients from other Chongqing hospitals. Third, the focus of this research is solely on the relationship between mortality, insurance type, and self-paying rate. Finally, active patient follow-up in this study may result in information bias. In future studies, outcome variables such as patient survival time and progression free survival time should be more detailed.

## Conclusions

According to the findings of this study, patients in southwest China who have a lower self-pay rate are at an increased risk of dying from cervical cancer. The reason may be that some treatments for cervical cancer are not covered by medical insurance. This phenomenon has been discovered by the Chinese medical insurance management department. In recent years, the National Health Insurance Bureau has reduced drug procurement costs and increased purchases through drug price negotiations and adjustments to reimbursement policies. More and more new anti-tumor drugs are added to the medical insurance catalog, which can reduce the economic pressure of patients. On the other hand, in order to decrease the prevalence of cervical cancer in China, high-risk groups should promote early cervical cancer screening and cervical cancer vaccination.

## Data availability statement

The original contributions presented in the study are included in the article/supplementary material, further inquiries can be directed to the corresponding authors.

## Ethics statement

The studies involving human participants were reviewed and approved by Affiliated Chongqing University Cancer Hospital Ethics Committee. The patients/participants provided their written informed consent to participate in this study.

## Author contributions

LY performed writing—original draft. HL performed formal analysis. LY and HL performed data curation. QZ and YW performed supervision and project administration. DZ, BW, XL, and QX performed investigation. All authors read and approved the final manuscript.
